# Ovarian Fluid Mediates the Temporal Decline in Sperm Viability in a Fish with Sperm Storage

**DOI:** 10.1371/journal.pone.0064431

**Published:** 2013-05-14

**Authors:** Clelia Gasparini, Jonathan P. Evans

**Affiliations:** Centre for Evolutionary Biology, School of Animal Biology (M092), University of Western Australia, Crawley, Australia; CNRS, France

## Abstract

A loss of sperm viability and functionality during sperm transfer and storage within the female reproductive tract can have important fitness implications by disrupting fertilization and impairing offspring development and survival. Consequently, mechanisms that mitigate the temporal decline in sperm function are likely to be important targets of selection. In many species, ovarian fluid is known to regulate and maintain sperm quality. In this paper, we use the guppy *Poecilia reticulata*, a highly polyandrous freshwater fish exhibiting internal fertilization and sperm storage, to determine whether ovarian fluid (OF) influences the decline in sperm viability (the proportion of live sperm in the ejaculate) over time and whether any observed effects depend on male sexual ornamentation. To address these questions we used a paired experimental design in which ejaculates from individual males were tested *in vitro* both in presence and absence of OF. Our results revealed that the temporal decline in sperm viability was significantly reduced in the presence of OF compared to a saline control. This finding raises the intriguing possibility that OF may play a role in mediating the decline in sperm quality due to the deleterious effects of sperm ageing, although other possible explanations for this observation are discussed. Interestingly, we also show that the age-related decline in sperm viability was contingent on male sexual ornamentation; males with relatively high levels of iridescence (indicating higher sexual attractiveness) exhibited a more pronounced decline in sperm viability over time than their less ornamented counterparts. This latter finding offers possible insights into the functional basis for the previously observed trade-off between these key components of pre- and postcopulatory sexual selection.

## Introduction

A number of studies have reported that ovarian fluid (OF) can have positive effects on the viability and/or motility of sperm (e.g. [1], [2]–[5]). These findings generate the readily testable prediction that OF may also serve a role in contributing to the survival of sperm over time, thus potentially mitigating the direct and indirect costs associated with a decline in sperm viability and functionality due to sperm ageing (for recent reviews see [6], [7]). In internally fertilizing species, studies investigating the effect of ovarian fluid (or more generally female-derived substances) on sperm survival are largely limited to insects (e.g. [4], [8]), and even in these systems such effects are poorly understood. Surprisingly, no study has explored patterns of sperm survival in any vertebrate with sperm storage. Yet studying the effect of short- and long-term storage on sperm survival not only increases our knowledge of the female's contribution to sperm survival, but may also offer insights into processes underlying cryptic female choice [9] when insemination and fertilization are temporally separated.

In this paper we examine whether OF mediates the temporal decline in sperm viability in guppies, *Poecilia reticulata*, a polyandrous freshwater poeciliid fish with internal fertilization. Throughout this paper, we refer to this temporal decline in sperm viability as either ‘sperm survival’ or ‘viability decline’. Female guppies are able to store sperm for extended periods, ranging from days to several months [10], [11]. Recent work has shown that OF plays an important role in regulating sperm quality (swimming velocity) in guppies, and this effect provides a mechanism by which females can differentially influence the fertilization success of sperm from different males when they compete to fertilize their eggs [3]. We assessed sperm viability (the proportion of live sperm per ejaculate) in fresh and experimentally aged sperm both in the presence and absence of ovarian fluid. Sperm viability is an important determinant of competitive fertilization success in poeciliid fishes [12] including the focal population of guppies used in the present study (Fitzpatrick, J.L. & Evans, J.P.; unpublished data). We used a highly controlled paired experimental design in which individual ejaculates were partitioned between treatments [3], thus controlling for the high level of variation that typically characterizes the viability of ejaculates (e.g. [13], [14]–[16]). This approach also allowed us to control the number of sperm that were exposed to each experimental treatment, and standardize experimental manipulation, as suggested by Holman [17]. Finally, we explored whether male colour ornamentation was associated with the decline in sperm viability. We know from previous work on guppies that males with relatively high levels of orange pigmentation produce both faster and more viable sperm than their less ornamented counterparts [18], [19]. However, there is also evidence from guppies that sperm quality is negatively (genetically) associated with the area of iridescent pigmentation, suggesting a possible trade-off between this component of male sexual ornamentation and sperm quality [16]. In the present study, we consider both colour traits (orange and iridescence) to determine whether any decline in sperm viability over time is dependent on male sexual ornamentation.

## Materials and Methods

### Ethics statement

This study was carried out in accordance with the Australian Code of Practice for the Care and Use of Animals for Scientific Purposes. The work was approved by the University of Western Australia's Animal Ethics Committee (permit number: RA/3/100/1050).

### Study species and its maintenance

The guppies used in this experiment were descendants of fish captured from Alligator Creek River in Queensland, Australia. These stocks were maintained in mixed-sex (approx. 1∶1 sex ratio) at 26±1°C and fed with *Artemia* nauplii until used in the experiment. Male guppies produce sperm packaged in bundles, which are transferred to females during consensual and forced matings [10]. Sperm remain quiescent inside these bundles but quickly become active inside the female's reproductive tract, typically reaching the ovary within 15 minutes of insemination [10]. Sperm not used immediately for fertilization are subsequently stored within the epithelial folds of the female's gonoduct and ovarian cavity [11].

### Experimental design

Adult females (*n* = 22) were randomly chosen from our stock population and OF was collected from each of these females by rinsing their gonoduct with a 0.9% NaCl solution (for details on the tecnique used see [3], [20]). Adult males (*n* = 22) were selected at random from stock aquaria and stripped of sperm using a standard procedure (e.g. [21]). Each male's ejaculate was then assigned randomly to one female's OF (ejaculates and OF samples were never reused across trials).

**Figure 1:**
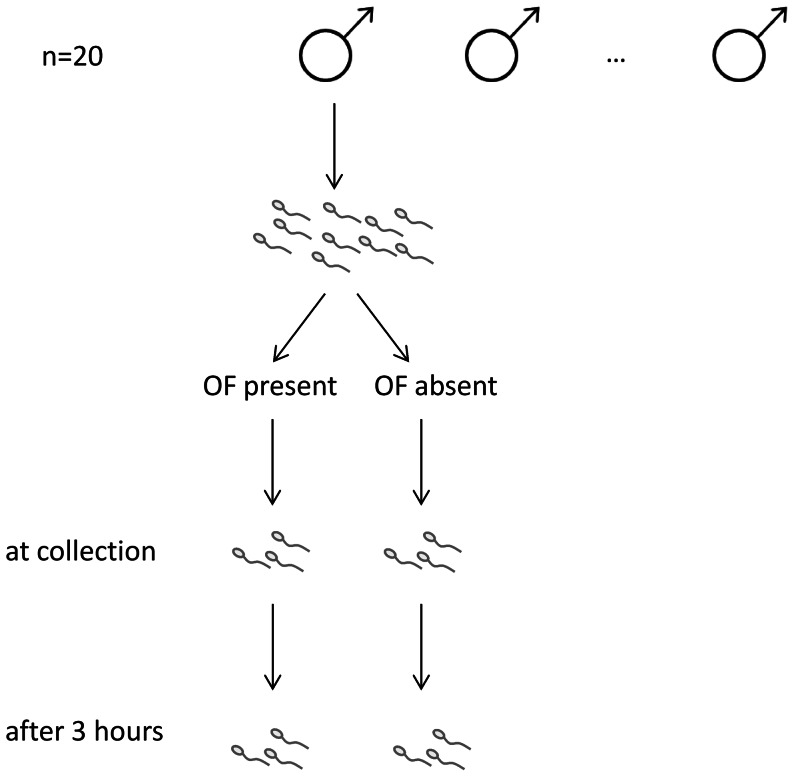
Schematic diagram showing the experimental design. Each ejaculate collected was split into two treatments (presence or absence of ovarian fluid, OF) and sperm viability was assessed twice in each treatment, initially at the time of collection and then after three hours of incubation.

For each replicate, we prepared two treatment solutions in replicate (i.e. two sub-samples of each solution, see Fig. 1). The first contained 40% of 150 mM KCl (to activate the sperm samples; [22]) and 60% NaCl solution containing ovarian fluid (hereafter ‘OF-present’) while the second contained 40% of 150 mM KCl and 60% NaCl solution (hereafter ‘OF-absent’). The two treatment solutions therefore differed only in the presence/absence of OF. KCl, present in both treatments, is routinely used to activate sperm in guppies and other poeciliid fishes (e.g. see [20], [22]–[24]). Activating sperm is particularly important in this internally fertilizing species in order to mimic the dissociation of sperm bundles and consequent activation process that takes place within 15 min of insemination [10], [11]. Importantly, we used the same proportion of ovarian fluid and KCl used in previous work on guppies to reveal the differential effects of ovarian fluid on sperm traits [3], [20]. After ejaculate collection, 10 individual sperm bundles (each containing ca. 22000 sperm [25]) were placed in the previously prepared KCl-NaCl solutions (i.e. either OF-present/absent). Sperm bundles were gently broken apart in their allotted treatments and immediately stained with a viability live-dead kit (L-7011, Molecular probes) to estimate sperm viability [26]. We assessed sperm viability again after three hours of incubation in the test solutions (i.e. in total we performed four viability assays for each male: two subsamples for each treatment, see Fig. 1). The proportion of live sperm (stained green) was assessed for approximately 100 sperm cells per trial (mean ± SE: 126.9±1.7, range: 98–164). The order in which sperm viability was scored across the two treatments was alternated to prevent bias.

### Male ornamentation

After sperm collection, the left side of each male was photographed using a Nikon D70 digital camera, and images were analysed for colour ornamentation using Image J software (available at http://rsb.info.nih.gov/ij/). We measured the area of orange (comprising yellow, orange and red coloration) and iridescent spots (comprising blue, green and white coloration) along with the body area. The relative area of coloured spots on the body was used in the analyses.

### Statistical analysis

Statistical analyses were performed using JMP v 9.0 (SAS Institute Inc.). Of the original 22 males, two did not produce sufficient numbers of intact sperm bundles at stripping and were therefore excluded from the analysis (i.e. final sample size  = 20). We initially used a paired *t*-test to test for the effect of treatment on sperm viability at time zero (freshly collected ejaculates) and after three hours of storage. We then tested for treatment and male ornamentation effects on sperm viability decline using a linear mixed-effects model in which the viability decline (subtracting the proportion of live sperm at time zero from the proportion of live sperm at three hours) was included as the dependent variable, treatment (presence or absence of OF) as a fixed factor, male coloration (proportion of iridescence and orange) as a covariate, and male identity as a random factor to account for the non-independence of data collected from the same male. Non-significant interaction terms were excluded from the final model. Proportion data were arcsine square-root transformed before being used in analyses. Means are reported with their associated standard errors (means ± SE).

## Results

**Figure 2:**
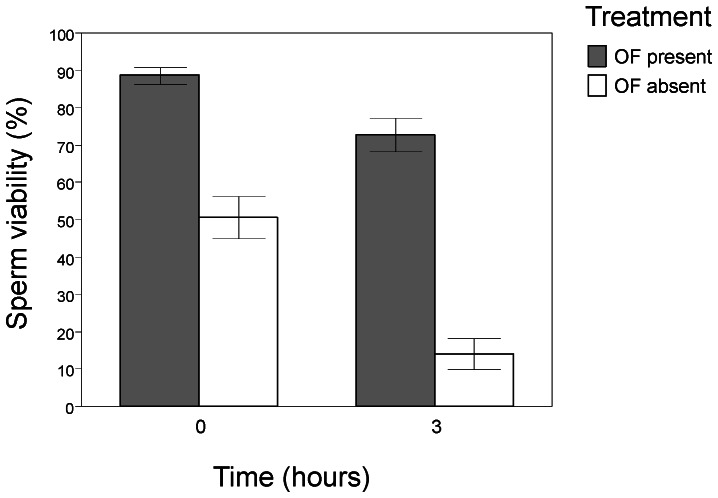
Sperm viability at time zero and after three hours. Bars are reported with standard errors.

**Figure 3:**
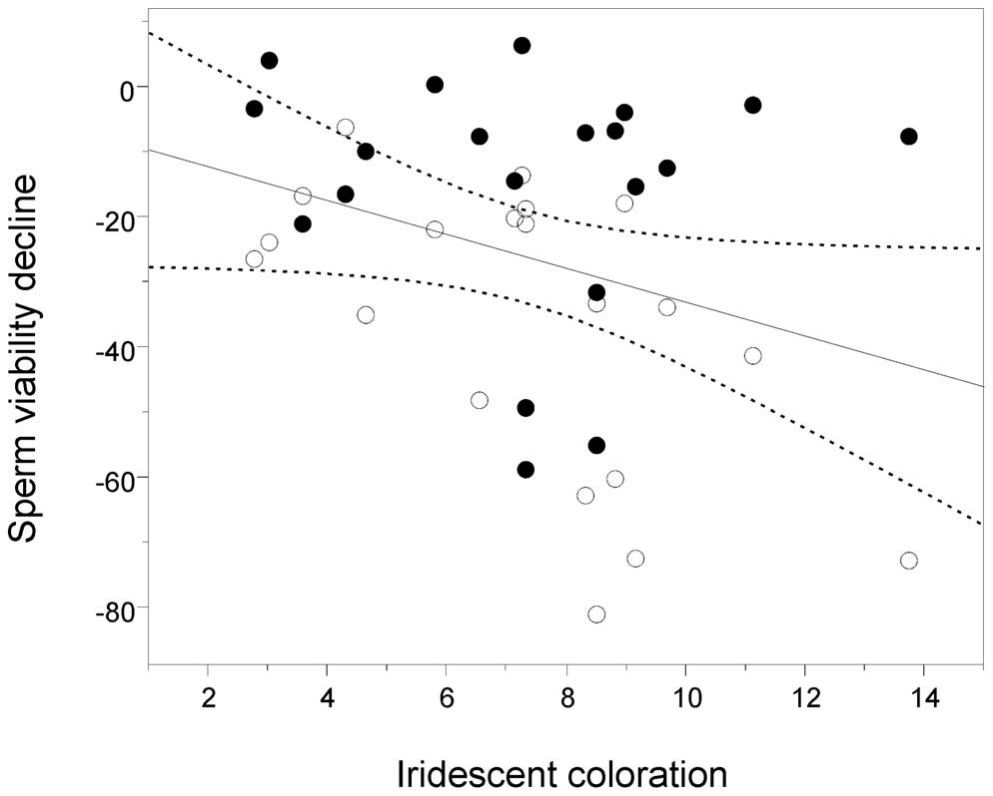
Decline of sperm viability in relation to male iridescent coloration. Sperm were incubated in presence (black circles) or in absence (empty circles) of ovarian fluid. Male iridescent coloration is showed in percentage.

The presence of OF enhanced sperm viability at both times (time at collection: t = 7.540, df  = 19, P<0.001, after 3 hours: t = 10.785, df  = 19, P<0.001, see Fig. 2). The mixed-effects model revealed significant effects of OF (presence/absence) and iridescent coloration, but no significant effect of orange coloration on the decline in sperm viability (see Table 1 for full model output). The decline in viability was less marked in sperm samples incubated in the presence of OF compared to those incubated without OF. Interestingly, the decline in sperm viability was more marked in males with higher levels of iridescence (see Fig. 3).

**Table 1:** Results from mixed-model analyses of sperm viability decline (difference in sperm viability between time of collection and after three hours of incubation in different solutions).

Variable	F	d.f.	P
Treatment (OF presence/absence)	9.817	19	0.006
Orange coloration	0.676	17	0.422
Iridescent coloration	6.133	17	0.024

## Discussion

We report the first evidence of a differential pattern of sperm viability decline according to the presence or absence of ovarian fluid during incubation, and the first evidence that female-derived fluids positively affect sperm survival in any vertebrate with sperm storage. Clearly the conclusions from the present study are limited to the effects of OF on the short-term decline in sperm viability *in vitro*, but they nevertheless raise a number of intriguing questions surrounding the possible adaptive functions of OF. One possibility is that OF helps mitigate the deleterious effects of sperm ageing during sperm storage. Sperm are typically more sensitive than other cells to the effects of ageing (reviewed in [6], [27], [28]) and it is now recognized that in addition to impairing fertilization success, sperm ageing can also have multiple deleterious indirect effects on female fitness by impairing the viability, development and condition of offspring [7], [28]–[30]. Given these effects, females are expected to evolve adaptations to avoid, or at least minimize, the effects of sperm ageing, particularly in species where sperm are stored for prolonged periods prior to fertilization [6], [31]. For example, females may benefit through increased fecundity by nurturing sperm, thus ensuring the availability of viable sperm over extended periods. Although female guppies are unlikely to be limited by the availability of sperm during a single reproductive event [32], brood size declines over successive broods [33], suggesting that maintaining a supply of viable sperm may be important for female fitness when the availability of mates is low. Clearly, such an adaptive role of OF depends both on an extrapolation of our findings to include beneficial effects of OF during long-term sperm storage, and on the assumption that OF mediates the temporal decline in sperm viability *in vivo*.

Other potential explanations for our findings do not invoke sperm ageing over prolonged periods. For example, females may use OF to nurture sperm, thereby ensuring that a high number of viable sperm successfully reach the site of sperm storage. Likewise, the process may also be male-driven. Sperm may be better adapted to survive within the female internal environment than to a ‘control’ saline solution due to the selective advantage of maximising the number of viable sperm that reach storage and/or the site of fertilisation, with possible consequences for sperm competitiveness. The observation that guppy sperm are able to utilise extracellular sugars [34] is consistent with both scenarios, although support for either depends on future studies that distinguish between short- and long-term effects of OF on the decline in sperm viability and those that quantify the ensuing implications for male and female fitness.

The role of OF in influencing sperm swimming behaviour has receiving increasing attention in recent years, especially in fishes. For example, OF has been show to extend sperm longevity (measured as the time that sperm were actively swimming) in brown trout, *Salmo trutta* [35], and Arctic charr, *Salvelinus alpinus* [2]. Ovarian fluid has also been shown to exert differential effects on patterns of sperm motility. For example, in the chinook salmon, *Oncorhynchus tshawytscha* [36], and the guppy [3], OF differentially regulates sperm swimming behaviour so that sperm from specific (compatible) males exhibit enhanced swimming performance compared to those from less compatible males. In the case of guppies, the differential effects of OF on sperm swimming velocity have direct fitness consequences, with OF mediating fertilization success in favour of unrelated males and thus serving as a mechanism of inbreeding avoidance [3]. In insects, cross-sectional sperm assays (i.e. those conducted at a particular point in time) also suggest an effect of female-derived fluids/secretions on sperm viability ([4], but see [8]) but these studies were not designed to explore temporal declines in sperm viability and the possible role that female-derived secretions might play in mitigating these effects.

Although sperm storage by females is widespread and has important evolutionary and ecological consequences [31], the mechanisms underlining the maintenance of viable sperm during prolonged storage are not fully understood. Nevertheless, secretions arising from female sperm storage organs (e.g. spermathecae) are known to contain various sugars, proteins and antioxidants that interact with sperm, and these substances are thought to have a positive effect on short- and long-term sperm survival during storage (e.g. [37], [38], [39]). In the honey bee, *Apis mellifera*, for example, spermathecal glands are the source of antioxidant enzymes that are thought to be involved in the long-term protection of sperm from oxidative stress [40]. These enzymes, in turn, are likely to account for the positive effect of spermathecal fluid on sperm viability in honey bees [4]. In the cricket, *Gryllus bimaculatus*, sperm stored in the spermatheca exhibit a marked reduction in metabolic rate and reactive oxygen species (ROS) production compared to freshly ejaculated sperm, suggesting that females play an important role in extending the lifespan and functionality of stored sperm in this species [41]. In guppies, the mechanisms that facilitate prolonged sperm storage have yet to be uncovered, although it is possible that the presence of glycogen in the ovarian epithelium and ovarian sugars [34] may serve to nourish sperm during storage [11].

We also explored the decline in sperm viability in relation to male sexual ornamentation to determine whether the rates of decline in sperm viability differ according to male phenotype. We found that males with relatively higher levels of iridescence had a more marked decline in sperm viability compared to less coloured males, which is consistent with previous work showing that iridescent coloration is negatively (genetically) correlated with sperm viability in this population [16]. Thus, a trade-off between investment in attracting mates (precopulatory sexual selection) and competing for fertilizations (postcopulatory sexual selection), which is predicted by sperm competition theory [42], [43], may be contingent on differential rates of sperm ageing among males differing in sexual attractiveness. Interestingly, even though not significant, a visual inspection of our results (Fig. 3) suggests that the relationship between the decline in sperm viability and male iridescent colouration was more prevalent in the OF-absent treatment compared to the OF-present treatment. This raises the intriguing possibility that OF may mitigate the trade-off between male sexual attractiveness and the decline in sperm viability. However, the interaction between treatment and iridescence was non-significant, and future work should explore potential differential effects of OF on sperm survival over longer periods to test this possibility.

Finally, our findings set the stage for future work that explores the possible role that OF-mediated declines in sperm viability play during sperm competition, where sperm from two or more males compete to fertilize a female's eggs [44]. Female guppies are highly polyandrous, but they also endure high rates of unsolicited (forced) mating attempts both within and outside their periods of sexual receptivity [45]. Thus, the potential of OF to differentially regulate the survival of sperm from different males, and hence the outcome of sperm competition, is likely to be adaptive for female guppies, especially where stored sperm may arise from unwanted forced matings. Our findings, in conjunction with recent studies revealing the OF's role in regulating sperm velocity and competitive fertilization success [3], [20], raise the intriguing possibility that OF, as a mediator of cryptic female choice, may enable females to bias paternity against sperm arising from forced matings. Our ongoing research specifically addresses this possibility by determining whether females have the capacity to differentially protect sperm during storage and thereby influence both the number and quality of stored sperm that compete for fertilization.
